# Women and HIV in Sub-Saharan Africa

**DOI:** 10.1186/1742-6405-10-30

**Published:** 2013-12-13

**Authors:** Gita Ramjee, Brodie Daniels

**Affiliations:** 1 HIV Prevention Research Unit, South African Medical Research Council, 123 Jan Hofmeyr Road, Westville, Durban 3629, South Africa; 2 London School of Hygiene and Tropical Medicines, Keppel Street, London WC1E, 7HT, UK

**Keywords:** HIV incidence, Sub-Saharan Africa, Adherence, Prevention

## Abstract

Thirty years since the discovery of HIV, the HIV pandemic in sub-Saharan Africa accounts for more than two thirds of the world’s HIV infections. Southern Africa remains the region most severely affected by the epidemic. Women continue to bear the brunt of the epidemic with young women infected almost ten years earlier compared to their male counterparts. Epidemiological evidence suggests unacceptably high HIV prevalence and incidence rates among women. A multitude of factors increase women’s vulnerability to HIV acquisition, including, biological, behavioral, socioeconomic, cultural and structural risks. There is no magic bullet and behavior alone is unlikely to change the course of the epidemic. Considerable progress has been made in biomedical, behavioral and structural strategies for HIV prevention with attendant challenges of developing appropriate HIV prevention packages which take into consideration the socioeconomic and cultural context of women in society at large.

## Introduction

Sub-Saharan Africa (SSA) remains the region most affected by the HIV epidemic. Almost three quarters (69%) of the 23.5 million people infected worldwide reside in this region [1]. Most countries in SSA report a generalized epidemic (infection rates of > 1%) with pockets of concentrated epidemics in key populations [1]. South Africa remains the country with over 6 million people reported to be infected with HIV [2]. Swaziland has the highest adult prevalence rate of 26.5% followed by South Africa, Namibia and Mozambique at 17.9%, 13.3% and 11.1% respectively [2]. Transmission is mainly through heterosexual sex, with women disproportionately infected compared to their male counterparts. It is reported that every minute one young woman becomes infected with HIV [3]. The purpose of this review is to discuss the HIV epidemic among women in SSA, associated risks for HIV acquisition, and to provide a brief update on HIV prevention options which may collectively impact on reducing incidence of HIV infection.

## HIV and women in Africa

In SSA, women bear the brunt of the HIV epidemic [2]. In 2011, 92% of pregnant women who were HIV positive were living in this region [4]. Most countries in SSA report the state of the epidemic based on their ante-natal surveillance data with limited data on HIV incidence due to the difficulty in accurately measuring HIV incidence rates. A number of studies conducted among non-pregnant women in parts of east and southern Africa suggest prevalence rates ranging from 14.5% (95% CI 11.2 – 18.4) in east Africa to 38.7% (95% CI 34.2 – 43.3) in Lusaka (Zambia) and 39.5% (95% CI 35.1 – 44.0) in Durban, South Africa [5]. There have been reports of a decline or stabilizing in HIV prevalence among women in southern Africa [6,7]. However, HIV incidence data from large scale clinical trials and cohort studies suggests that the low prevalence masks high HIV incidence rates [8-11]. Risk factor analysis of these cohorts of women from various HIV prevention trials suggest that being under the age of 25 years, having had one sexually transmitted infection (STI) in the past and being unmarried were significantly associated with high risk of HIV seroconversion [12,13]. Rehle *et al.* substantiated these results in the 2013 Human Sciences Research Council (HSRC) household survey which showed that being young and unmarried increased the risk of HIV acquisition among South African women [14].

## Factors that increase women’s vulnerability to HIV in Africa

The disproportionate impact of the HIV epidemic on women can be attributable to several factors including biological, social, behavioral, cultural, economic and structural. In SSA a combination of these factors has led to the disparate increase in HIV infection rates among women compared to their male counterparts [15].

## Biological risk factors

Women are at a greater physiological risk of contracting HIV than men. This is in part because women have a greater mucosal surface area exposed to pathogens and infectious fluid for longer periods during sexual intercourse and are likely to face increased tissue injury. Young women are at particularly high risk due to cervical ectopy which facilitates greater exposure of target cells to trauma and pathogens in the vagina [16]. It has been well documented that STIs increase the risk of HIV acquisition [17]. For women, the risk is increased due to difficulty in diagnosing STIs which are often asymptomatic in presentation [18], thus making treatment difficult [16]. A study by Ramjee *et al.*[5] determined the prevalence of STIs (such as *Neisseria Gonorrhea, Chlamydia Trachomatis, Syphilis*, and *Trichomonas Vaginalis)* at 4 clinical trials sites namely Durban and Hlabisa (South Africa), Lusaka (Zambia) and Moshi (Tanzania). The study showed that women at the South African sites were 3 times more likely to acquire an STI than participants at the Tanzanian and Zambian sites. Studies on HIV/STI “hot spots” among women in communities in the greater Durban area in the province of KwaZulu-Natal (South Africa) theorize an overlap of prevalence and incidence of these infections, suggesting increased risk of HIV acquisition in STI/HIV “hot spot” areas within these communities [19].

Hormones such as progesterone are reported to be playing a role in a woman’s biological vulnerability to HIV infection. Observational evidence suggests that progesterone containing injectable contraception depot medroxyprogesterone acetate (DMPA) may be putting women at higher risk of HIV acquisition [20-23]. There is however some conflicting studies which have not observed this risk [24-26]. Injectable contraception is one of the most widely used contraceptive methods of choice among women in SSA, the use of which is on the rise in certain countries such as Kenya, Madagascar and Malawi [27]. Based on conflicting evidence on the role of DMPA on HIV acquisition, the World Health Organization (WHO) released a statement recommending that women on hormonal injectable progestin contraception should be counseled to use condoms in conjunction with pregnancy prevention methods to prevent HIV acquisition [28].

Studies in Uganda, Rwanda and Zimbabwe have shown that pregnant women are at a higher risk of HIV infection than lactating or other women, possibly due to physiological changes that a woman undergoes during pregnancy [29]. High levels of oestrogen and progesterone either during pregnancy or from exogenous sources could cause changes in the structure of the genital mucosa or cause immunological changes, such as an increase in mucosal lymphoid aggregates or hormone-induced overexpression of co-receptors associated with HIV infection. Increased levels of oestrogen are also associated with cervical ectopy in young women which in turn increases risk to HIV infection [29].

Supporting evidence suggests that women have a window of vulnerability approximately seven to ten days after ovulation in their menstrual cycle in which the potential for viral infectivity in the female reproductive tract is increased. This is due to the suppressing influence of sex hormones on the innate, humoral and cell-mediated immune systems. This takes place in the upper and lower female reproductive tract, and overlaps with the upregulation of co-receptors for HIV uptake and the recruitment of potentially infectable cells [30].

## Contextual variability in risk

For women in SSA, probabilities of HIV exposure, acquisition and transmission is influenced by a range of factors such as infectiousness of the male partner according to the stage of the disease, or presence of ulcers in the male partner in the event of unprotected sexual intercourse. At a broader societal level, several factors influence the scale and rate of the epidemic spread such as overall HIV and STI prevalence, sexual practices, marriage and other cultural norms including structural factors not in control of the individual.

The dominant patriarchal culture and society in Africa exacerbates women’s inferiority and their disparate health status. Here, the needs and desires of women are not considered significant and often women play no part in sexual decision making, nor are they allowed to express their sexuality [31,32]. There may be violent consequences if a woman were to take the initiative in sex, suggest condom use or refuse sexual advances [31]. In certain cultures men are regarded as the heads of the family, decision-makers and the ones who control resources and finances while women are expected to respect their husbands, accept polygamous relationships and fulfill family and community tasks [33]. Cultural expectations of masculinity encourage men to assume the patriarchal attitude that wives, partners and daughters are the possessions of men, and most husbands expect or demand their conjugal ‘rights’ [33]. One cultural practice that perpetuates the idea that a woman is her husband’s property is “bride payments”, where the bride’s family receives financial compensation from the potential husband. Due to the issue of bride payment or “lobola” many men in relationships are unable to “pay” for the woman they would like to marry resulting in many men moving from one relationship to another leaving the woman (more likely with his children) to economically fend for herself and the children [34,35]. Women then look for other partners resulting in a complex web of cultural practice and multiple partnerships placing both men and women at high risk of HIV infection.

In countries such as Malawi, Kenya, Zambia, and Botswana, it is widely believed that a widow becomes unclean after her late husband’s burial ceremonies. In cleansing rituals, an unprotected sexual act is believed to purify the recipient through the semen entering the woman’s body [36]. Community elders identify a man with whom the widow has to have sex; someone who has often had several sexual partners in the process of cleansing others. These practices often put widowed women at greater risk of HIV and other STI infection [36]. The ritual is also practiced in other situations, such as after a birth the mother, regardless of marital status, has unprotected sex with a man believing the act will cleanse the baby and encourage health; after miscarriage; and where a man has acquired a boat for fishing, a woman has sex with the man to cleanse away evil spirits which may capsize the boat [36]. Sexual cleansing is also believed to ‘cleanse’ people living with HIV; in some African countries it is believed that sex with a virgin can cure the disease [16,36].

The practice of “dry sex” is reportedly high in some southern African countries [36]. This practice involves inserting drying agents into the vagina (herbs, a dry cloth, or even chemicals) to produce the required tight, dry and “hot” vagina. This practice is believed to provide heightened sexual pleasure for the male partner. For women, it causes friction and possible tearing of delicate vaginal mucosal lining, increasing the risk of HIV acquisition. Use of harmful chemicals can cause inflammation and lesions and alter the natural vaginal pH level, potentially increasing women’s risk of contracting HIV. However, no studies have confirmed the role of “dry sex” in HIV acquisition [29,36,37]. Deeply entrenched traditional and cultural practices; often interwoven with religious beliefs make change in behaviour very difficult and complex [36].

Virginity testing, (a physical examination of girls and women to determine whether the hymen is unbroken), is now widely regarded as a human rights abuse. Although virginity testing was a common traditional practice among unmarried couples in South Africa and Uganda, it was, until recently, no longer commonplace. However, in an attempt to promote abstinence among girls, this practice has seen resurgence [36]. A concern with this ‘forced abstinence’ is that girls and unmarried women may engage in risky anal sex to avoid failing virginity tests, placing them at even higher risk of HIV acquisition [36].

In many African countries, young girls are encouraged or forced to marry older men [36,38] which can make young girls vulnerable to HIV infection in several ways. The change from virginity to frequent unprotected sex increases their risk of HIV infection. Even if they were sexually active prior to marriage, marriage usually drastically increases the frequency of unprotected sex [39]. In Malawi, for example, consent to sex is assumed within a marriage and therefore marital rape does not exist [31,36]. Many African countries condone polygamy [38], which means that an adolescent bride is likely to be a second or third wife [39]. Women who marry as adolescents are also more likely to receive less formal education and have less exposure to HIV prevention messages [38,39]. A study in Durban, South Africa, demonstrated that if age of sexual debut was 15 or younger, the women were more likely to be HIV positive compared to women who had their sexual debut at the age of 21 or older (Hazard ratio of 1.97; 95% CI 1.07 to 3.63, p = 0.029) [40].

## Socioeconomic vulnerabilities

In the last two decades, nearly all SSA countries have faced slowing economic growth which has influenced spending on social services [32]. This has further impoverished African populations, with increases in unemployment rates and the decrease in provision of social services, including education and health services. The deterioration of education, health, and other social services implies a loss of opportunities for HIV prevention [32], particularly in women.

Poverty is another driving force of HIV transmission in women [33]. Evidence from SSA, Asia and Latin America demonstrates that greater national-level income inequality is associated with higher HIV prevalence rates [41,42]. Low economic status has been associated with earlier sexual experience, lower condom use at last sex act, having multiple sex partners, increased chances that the first sex act is non-consensual, and a greater likelihood of having had transactional sex or physically forced sex [15]. Many women resort to transactional sex to sustain their livelihoods and young girls are often coerced into sexual activities with older men to survive [32,33]. Cash transfers among secondary school-aged young women in Malawi showed that such transfers can encourage women to reduce their risky sexual behaviour, and resulted in decreased teenage pregnancy, in addition to lower self-reported sexual activity [33]. In conditions of poverty, HIV risk becomes a low priority among people’s daily concerns. Young people growing up in poor conditions have little access to schooling and therefore few prospects for their future. Sex becomes a way to pass time due to a lack of recreational facilities [32].

Transactional sex (sex for money) is common in SSA. Many women engage in sex work which is defined as the provision of sexual services in exchange for money, goods, or other benefits [43]. Globally, female sex workers (FSWs) are at high risk of HIV infection and are 13.5 times more likely to be living with HIV than other women [2]. An estimated fifteen percent of HIV in the general female adult population is attributable to (unsafe) female sex work. The region with the highest attributable fraction is SSA with 98,000 HIV-related deaths [44] compared to an estimated 106,000 deaths worldwide which are a result of female sex work [45]. FSW is an important contributor to HIV transmission and the global HIV burden. Studies from South Africa and Kenya suggest sex workers engage in high risk behavior such as dry sex [46,47] and anal sex (fetching a high price) which further increase their risk.

## Behavioral vulnerabilities

It is well documented that behavioral change can lead to a decrease in HIV acquisition [48]. These include use of methods such as abstinence or delaying sexual debut, condoms, safe sex, monogamy, reduction in number of partners, voluntary counseling and testing etc. However abstinence is not an option for many women. A South African household survey showed that 7.8% of women aged 15–24 years had had sexual intercourse by the age of 14 [49]. An association has been shown between early age of sexual debut and ensuing risky sexual behaviors [50], such as having multiple partners and decreased contraceptive and condom use [51,52]. Studies in Africa demonstrated a correlation between having sex at an early age and the HIV incidence [53,54]. It is thought that the delay of sexual debut may have been one of the crucial changes in behavior which led to a decline in HIV infection in Uganda [40,55,56].

Concurrent partnerships are powerful transmitters of HIV in the community [57]. Concurrent sexual partnerships, with two or more partners, are common practices in Africa. Partner reduction and fidelity appear to be the main behavioral changes accountable for the decrease in Kenya’s HIV infection rates. This fall in HIV infection rates as a result of partner reduction has also been observed Zimbabwe, Côte d’Ivoire, Malawi and Ethiopia [58].

The vulnerability to HIV infection increases by engaging in unprotected anal sex by 13 fold compared to oral sex [59]. This is primarily due to the fragility of the rectal mucosa [29]. Although it is difficult to ascertain the percentage of women who participate in heterosexual anal sex in Africa, some studies and surveys have shown that between five [60] and twelve per cent [61] of women in the general community in South Africa report engaging in anal sex. However, this practice could be as high as forty per cent in female sex workers [59]. Effective HIV prevention measures exist and have been successfully targeted at key populations in many settings. It is however difficult to attract this high risk population group due to the different settings in which the FSWs conduct their business. Regular surveillance, prevention and treatment of HIV among FSWs would benefit this often neglected vulnerable group and the general population as a whole [2].

Alcohol abuse in much of Africa is characterized by irregular episodes of heavy drinking, frequently in the form of weekend bingeing [29]. These drinking patterns may have independent effects on sexual decision-making, and on condom-negotiation skills and correct condom use. Studies have demonstrated that women with heavy episodic drinking patterns are more prone to use condoms inconsistently and incorrectly, experience sexual violence; and acquire an STI, including HIV [29,62].

## Structural vulnerabilities

Many structural vulnerabilities place women at risk including gender inequality and gender based violence (GBV) [63], migration and health seeking related stigma [64]. Gender inequality is created and perpetuated in part by social norms that demand culturally appropriate roles and conduct for men and women [65]. Hierarchical gender roles such as notions of male sexual entitlement, the low social value and power of women, and ideas of manhood linked to the control of women result in lower levels of education among women; few public roles for women; the lack of family, social and legal support for women; and the lack of economic power for women [65,66]. Institutionalized economic inequalities keep money, land, and other resources out of women’s reach, causing women to be financially dependent on men, more likely to practice transactional sex, less likely to be able to negotiate safe sex or condom use with a partner, and more at risk of violence [6,16,49,65,67].

Gender norms promote multiple concurrent sexual partners for men while women are expected to be monogamous and unquestioning of their partner’s behavior [49,65]. However, recent data from several African studies show that many women are almost as likely as men to bring HIV into the partnership [68]. Sexual negotiation or refusal by a woman may result in suspicions of infidelity and result in intimate partner violence [49], which is often tolerated by these societies [65]. Women’s sexual subordination exposes them to elevated reproductive health risks including maternal mortality, unsafe abortion, coerced sex and rape, STIs and HIV [63].

Violence against women is a global problem and a well-recognized risk factor for HIV and other STIs [16,63,69]. In certain countries, over 50% of women report physical assault by an intimate partner, with one third to one half of physically abused women also reporting sexual coercion [65,70]. Abusive men are more likely to have additional sexual partners unknown to their wives [69]. Experiences of sexual violence have also been associated with drug and alcohol use, early sexual debut, having multiple sexual partners, trading sex for money and drugs and less contraceptive use [66]. Women who live in poverty, younger women and uneducated women are especially affected by intimate partner violence [70-72]. A South African study found that 12% of new HIV infections in women were attributable to intimate partner violence [71], while GBV increased the risk of HIV infection by 55% in a Ugandan study [73].

Discrimination is often based on gender, race/ethnicity, sexual orientation, and HIV status [74]. Stigma and fear of status disclosure has been a large factor in individuals seeking HIV testing and subsequent treatment and care [33,75]. Stigma can occur at different levels, including community, interpersonal, legislative and institutional (e.g. workplaces, schools and health facilities) levels [75]. Women often suffer the heaviest burden of HIV stigma and discrimination, as they are often expected to uphold the moral traditions of their societies; being HIV infected is considered evidence that they have failed in this regard [33,74]. HIV positive women experience discrimination, stigma and other human rights violations within families and communities, by legal and social services, in health-care settings, and in their work environment. Health-care settings often refuse to provide information or provide the wrong information on HIV prevention and treatment, sexual and reproductive health, and family planning [33]. HIV positive women have also experienced the denial of services, lack of confidentiality, harsh and judgmental treatment, and lack of informed consent [33]. As a result of stigma, women are often reluctant to seek HIV testing and are not empowered to enact HIV prevention [74].

Migration trends in Africa vary in terms of their spatial, temporal and social characteristics. Many individuals migrate due to better job prospects or are forced to migrate due to political instability, war and famine [32]. Worldwide, mobile populations have higher HIV infection rates than non-mobile populations irrespective of HIV prevalence in the origin or destination location [74]. Mobility per se can encourage high risk sexual behavior and more than often migrants’ social networking creates opportunities for sexual networking. Women are becoming more mobile and are migrating for economic survival. However in many cases, lack of education often restricts them to unskilled jobs such as informal trading, commercial sex work and domestic work among others. A study conducted in South Africa among migrant and non-migrant women showed that migrant women were older, more likely to be married and had more than one sexual partner and were less likely to use condoms compared to non-migrant women. Migrant women were 1.6 times more likely to be infected with HIV than non-migrants [76].

Urbanization has also fuelled the rapid spread of HIV. In SSA, urban HIV prevalence is higher than rural populations [32]. Urbanization replaces the traditional village norms for an urban modern culture with fewer restrictions on sexual behaviour and marriage. Additionally, the loss of culture and social networks are related to social problems such as drug abuse, which fosters high-risk behaviour [32]. Political conflicts and war are commonplace in Africa and affects the risk of HIV/AIDS by interrupting normal social and risk networks, increasing poverty and social instability and weakening or destroying medical infrastructure [74]. Military conflict can cause changes in risk behavior, and war or civil strife often cause massive displacements of people [74], interrupt social cohesion and relationships, and encourages promiscuity and commercial sex; forced migration of people from low HIV prevalence areas to areas of high HIV prevalence [32]. Women who are temporarily displaced or in transit as a result of war or violence are more likely to report having received goods or money for sex [62].

## HIV prevention strategies targeted at women

Given the magnitude of women’s vulnerability to HIV acquisition as described above, it is not surprising that there is a worldwide call to address the high HIV infection rates reported among women in SSA and elsewhere. Biomedical, behavioural and structural interventions have been investigated to reduce women’s vulnerability to HIV acquisition but also HIV incidence in general.

## Biomedical interventions

Male condoms in general remain the most effective method of HIV prevention. However condom use or negotiation thereof is not always in the woman’s hands. Although female condoms are available, the use still requires negotiation. Thus female-initiated prevention methods have been investigated.

Microbicides are products with anti-HIV properties developed in numerous formulations for prevention of HIV. Microbicides were initially developed for vaginal application but are now also tested for rectal application [77]. Over the past decade several microbicides with varying actions such as membrane disruption activity (surfactants), prevention of virus attachment to target cells (polyanions), and buffering agents have been tested with limited success primarily due to lack of specificity against HIV and poor adherence to interventions by women [10,11,78-80]. More recently, products containing anti-retorviral (ARV) agents commonly used in HIV therapeutics were tested. A study of tenofovir based vaginal gel in South Africa showed a 39% (95% CI 6 to 60) reduction in HIV acquisition [81]. The greater the adherence to the product, the better was the efficacy in HIV prevention. Unfortunately the results were not replicated in a larger study conducted in several countries in SSA using the same gel [82]. The main reason for lack of efficacy was poor adherence. Further research is being undertaken to ascertain women's reluctance to use such interventions given the high risk of HIV acquisition in this region. A study with a long-acting vaginal ring with a drug called Dapivirine is currently ongoing (ASPIRE) [83]. Lessons from microbicide trials suggest the need to better understand social, cultural and traditional contexts of women in Africa while developing interventions for HIV prevention.

Success with research among HIV discordant couples showed a decrease in HIV acquisition when a negative partner in a dicordant partnership was provided with oral pre-exposure prophylaxis (PrEP). Partners taking oral tenofovir or tenofovir plus emtricitibine (Truvada) as prophlaxis had a reduced rate of HIV acquisition 67% (95% CI 44 to 81) and 75% (95% CI 55 to 87) respectively [84]. The higher the adherence the greater the efficacy. Unfortunately a study among women in east and southern Africa testing the same drug, Truvada, for HIV prevention did not show any efficacy in preventing HIV due to poor adherence to the study product [85].

Treatment with ARV for prevention (TasP) has been reported to be highly efficacious (96%) among both men and women in a discordant couple relationship where the positive partner was provided ARV treatment to reduce HIV transmission to the negative partner [86]. Thefore it appears that when both sexual partners have the knowledge of their HIV status and are targeted together for HIV prevention the intervention is likely to be aceptable and used.

## Behavioral interventions

HIV counseling and testing is recognized as a critical component for all HIV prevention interventions. Testing provides the opportunity to identify people who are HIV positive and in need for treatment and care and can also identify people who are HIV negative and referred for HIV prevention and care. Regular HIV testing will be critical for prevention interventions using PrEP. HIV counseling and testing not only allows women, couples, and families to learn their HIV status and obtain personalized risk reduction counseling and further care appropriate to their status, but can also assist communities in addressing stigma and discrimination associated with HIV/ AIDS [87,88].

Peer education interventions in developing countries have shown a significant increase in HIV knowledge and increased condom use. However, these programs have shown only a moderate improvement of behavioral outcomes, with no significant effect on biological outcomes [89]. Kenyan peer educators were able to increase protected sex among sex workers by providing condoms; facilitating HIV testing, treatment and care facilities and providing STI and HIV education [90]. In South Africa, the Stepping Stones program used participatory learning approaches to build communication skills, knowledge, and risk awareness. The program saw a 33% reduction in Herpes simplex virus-2 (HSV-2) incidence and improved several reported risk behaviors in men, such as transactional sex and intimate partner violence, although no decrease in HIV incidence was noted [91]. In developing countries, many mass media interventions have targeted changing HIV-related knowledge, behaviors and attitudes. At least half of these studies which reported outcomes such as condom use, reduction in risky sexual behavior, awareness of the modes of HIV transmission, HIV risk perception, ability to negotiate condom use and sexual abstinence showed a positive impact on the knowledge of HIV transmission and the decrease of risky sexual behavior [92]. Zambia has implemented several health communication programs focusing on HIV prevention and people who had been exposed to these radio and television programs were more likely to have ever used a condom [93]. In Nigeria, mass-media campaigns via television, radio and printed advertisements to increase the use of family planning, HIV/AIDS services and child survival demonstrated that people were twice as likely to know that HIV risk is reduced by condoms and one and a half times as likely to discuss HIV with their partner if they had high exposure to the program [94].

It is widely known that women who have an early sexual debut participate in risky sexual behavior, such as having multiple partners, higher STI incidence and decreased condom and contraceptive use [51,52]. High level governmental encouragement of the delay of sexual debut could have contributed to the decline in Uganda’s HIV infection [55,56].

Concurrent sexual partnerships are recognized as being significantly responsible for the transmission of STIs, particularly heterosexual HIV transmission. Evidence implies that concurrent partnerships can increase the size of an HIV epidemic, the speed at which it infects a population and its persistence within a population [57]. Uganda adopted a “Zero Grazing” campaign in the 1980’s, where having concurrent partners was discouraged. This campaign showed a 50% reduction in the number of people reporting casual and multiple partners between 1989 and 1995 [58,95]. It is also believed that partner reduction and fidelity are the main behavioral changes responsible for the decline in Kenya’s HIV infection rates. Similar behavior changes have been reported in Cote d’Ivoire, Zimbabwe, Ethiopia and Malawi which may be responsible for their decrease in HIV prevalence [58].

## Structural interventions

Structural interventions attempt to change the social norms that worsen HIV vulnerability. The South African IMAGE project sought to decrease gender based HIV vulnerabilities such as sexual violence, women’s lack of knowledge about HIV and its transmission and women’s economic dependency on men. The study partnered with a local microfinance institution to permit women to participate in microenterprises, while providing them with HIV education and a forum to discuss action against gender-based violence. These women improved their household wellbeing and empowerment, in addition to the significant reduction in gender-based violence [64].

Microfinance programs attempt to ease poverty by offering access to credit, savings or business skills. In South Africa, a study using an integrated curriculum of gender equity, anti-violence work and HIV/AIDS education together with an existing microfinance program demonstrated higher levels of HIV-related communication by women in addition to a 55% reduction in domestic violence. Women in this study were less likely to have had unprotected sex at their last sex act and more likely to have accessed HIV Counselling and Testing [96]. In Malawi, girls receiving cash transfers had a lower prevalence of HIV and HSV-2 than those who were not because of less sexual activity, delayed sexual debut, having younger and fewer partners and decreased transactional sex [88]. A Tanzanian trial suggested a 25% lower incidence of STIs in both women and men receiving monetary incentives [88]. A Kenyan project provided HIV and safe sex education, small business management training and startup funds to women engaged in sex work. The project showed an increased sense of pride and wellbeing, a decrease in the number of sexual partners and an increase in condom use [97]. In response to the increase in HIV incidence caused by migration, a project in Tanzania distributed and promoted condom use, raised HIV awareness, encouraged reduction in the overall number of sexual partners and encouraged peer-based STI/HIV education among truck drivers. The number of condoms distributed increased by 100 000 and HIV awareness among truck drivers increased [97].

Education reduces risk of HIV infection, boosts self-esteem, and provides alternatives for earning a livelihood [50]. Communities and individuals with greater school education may be at decreased risk of HIV infection due to the adoption of protective lifestyles and behaviour [98]. People with little or no education have limited access to safe-sex information. For example, condom use is associated with higher education levels [32]. Keeping young girls in school has also showed a reduction in their risk of contracting HIV. Studies show that the expansion of primary and secondary schooling for girls in southern Africa will help reduce their vulnerability to HIV [99].

Policy structural factors play a major role in HIV transmission and Uganda is the best example of successful policy changes and the link to decreased HIV transmission. The Ugandan government committed itself to extensive training of trainers, mass media, health education campaigns, countrywide messaging and district mobilization causing changes in sexual behavior, social norms and HIV prevalence [100]. Research in Uganda has demonstrated the connection between a country’s government recognition of the importance of HIV and the subsequent development of better government interventions to HIV [97].

## Conclusion

Convincing evidence from biological, epidemiological, sociocultural and structural challenges identify women’s vulnerability to HIV infection in SSA. The evidence shows that women in SSA are at higher risk of HIV infection compared to their male counterparts. Through gender power dynamics, women are embedded in relationships which increase their risk even more. Other than personal behavior, several other issues such as structural influences are identified which are beyond women's control and are likely to affect women globally. Given the unacceptably high prevalence and incidence rates of HIV in young women in southern Africa, every effort to mitigate this risk is urgently needed with a combination of biomedical, behavioural and structural interventions targeted not only to the women but their sexual partner(s) as well. Recent advances in the HIV prevention field need to be stepped up by implementing appropriate HIV prevention packages taking into consideration the cultural environment, biological, socio-economic and structural risks facing women.

## Competing interests

The authors declare that they have no competing interests.

## Authors’ contributions

Both GR and BD contributed to the drafting of the manuscript. Both authors read and approved the final manuscript.

## References

[B1] UNAIDS Report on the Global AIDS Epidemic 2012 Geneva, Switzerland: Joint United Nations Program on HIV/AIDS (UNAIDS)

[B2] UNAIDS Global Report on the Global AIDS epidemic 2013 Geneva, Switzerland: Joint United Nations Program on HIV/AIDS (UNAIDS)

[B3] UNAIDS Every minute, a young woman is infected with HIV 2012 Geneva, Switzerland: Joint United Nations Program on HIV/AIDS (UNAIDS)

[B4] UNAIDS World AIDS Day Report 2012 Geneva: UNAIDS

[B5] Ramjee G The value of site preparedness studies for future implementation of phase 2/IIb/III HIV prevention trials: experience from the HPTN 055 study J Acquir Immune Defic Syndr 2008 47 1 93 100 10.1097/QAI.0b013e31815c71f7 10.1097/QAI.0b013e31815c71f7 17984760

[B6] UNAIDS How Africa turned AIDS around 2013

[B7] Rehle TM A decline in new HIV infections in South Africa: estimating HIV incidence from three national HIV surveys in 2002, 2005 and 2008 PloS One 2010 5 6 e11094 10.1371/journal.pone.0011094 20559425 PMC2885415

[B8] Karim QA Stabilizing HIV prevalence masks high HIV incidence rates amongst rural and urban women in KwaZulu-Natal, South Africa Int J Epidemiol 2011 40 4 922 930 10.1093/ije/dyq176 21047913 PMC3156366

[B9] Ramjee G Zero new infections: a myth or reality, in 6th SA AIDS conference 2013 Durban, South Africa

[B10] Karim SSA Safety and effectiveness of BufferGel and 0.5% PRO2000 gel for the prevention of HIV infection in women AIDS (London, England) 2011 25 7 957 10.1097/QAD.0b013e32834541d9 PMC3083640 21330907

[B11] McCormack S PRO2000 vaginal gel for prevention of HIV-1 infection (Microbicides Development Programme 301): a phase 3, randomised, double-blind, parallel-group trial Lancet 2010 376 9749 1329 1337 10.1016/S0140-6736(10)61086-0 20851460 PMC2956883

[B12] Wand H Ramjee G Assessing and evaluating the combined impact of behavioural and biological risk factors for HIV seroconversion in a cohort of South African women AIDS Care 2012 24 9 1155 1162 10.1080/09540121.2012.687820 22709144

[B13] Wand H Whitaker C Ramjee G Geoadditive models to assess spatial variation of HIV infections among women in Local communities of Durban, South Africa Int J Health Geogr 2011 10 1 28 10.1186/1476-072X-10-28 21496324 PMC3098769

[B14] Rehle T Shisana O HSRC National Survey 6th SA AIDS Conference 2013 Durban

[B15] Mabala R From HIV prevention to HIV protection: addressing the vulnerability of girls and young women in urban areas Environ Urban 2006 18 2 407 432 10.1177/0956247806069624

[B16] Ackermann L Klerk GW Social factors that make South African women vulnerable to HIV infection Health Care Women Int 2002 23 2 163 172 10.1080/073993302753429031 11868963

[B17] Kalichman SC Pellowski J Turner C Prevalence of sexually transmitted co-infections in people living with HIV/AIDS: systematic review with implications for using HIV treatments for prevention Sex Transm Infect 2011 87 3 183 190 10.1136/sti.2010.047514 21330572 PMC4317792

[B18] Connolly CA Incidence of sexually transmitted infections among HIV-positive sex workers in KwaZulu-Natal, South Africa Sex Transm Dis 2002 29 11 721 724 10.1097/00007435-200211000-00017 12438911

[B19] Ramjee G Wand H Geographical clustering of high risk sexual behaviors in “Hot-spots” for HIV and sexually transmitted infections in Kwazulu-Natal, South Africa AIDS Behav 2013 1 6 DOI: 10.1007/s10461-013-0578-x 23934268 10.1007/s10461-013-0578-x PMC3905176

[B20] Baeten JM Hormonal contraceptive use, herpes simplex virus infection, and risk of HIV-1 acquisition among Kenyan women Aids 2007 21 13 1771 1777 10.1097/QAD.0b013e328270388a 17690576

[B21] Ramjee G Wand H Population-level impact of hormonal contraception on incidence of HIV infection and pregnancy in women in Durban, South Africa Bull World Health Organ 2012 90 10 748 755 10.2471/BLT.12.105700 23109742 PMC3471058

[B22] Heffron R Use of hormonal contraceptives and risk of HIV-1 transmission: a prospective cohort study Lancet Inf Dis 2012 12 1 19 26 10.1016/S1473-3099(11)70247-X PMC3266951 21975269

[B23] Wand H Ramjee G The effects of injectable hormonal contraceptives on HIV seroconversion and on sexually transmitted infections Aids 2012 26 3 375 380 10.1097/QAD.0b013e32834f990f 22156970

[B24] Kleinschmidt I Injectable progestin contraceptive use and risk of HIV infection in a South African family planning cohort Contraception 2007 75 6 461 467 10.1016/j.contraception.2007.02.002 17519153

[B25] Myer L Prospective study of hormonal contraception and women’s risk of HIV infection in South Africa Int J Epidemiol 2007 36 1 166 174 10.1093/ije/dyl251 17175547

[B26] Kiddugavu M Hormonal contraceptive use and HIV-1 infection in a population-based cohort in Rakai, Uganda Aids 2003 17 2 233 240 10.1097/00002030-200301240-00014 12545084

[B27] Adetunji J Rising popularity of injectable contraceptives in sub-Saharan Africa African Popul Stud 2011 25 2 587 604

[B28] Organization WH Hormonal contraception and HIV: technical statement 2012 Geneva, Switzerland: World Health Organization 23586117

[B29] Chersich MF Rees HV Vulnerability of women in southern Africa to infection with HIV: biological determinants and priority health sector interventions Aids 2008 22 S27 S40 19033753 10.1097/01.aids.0000341775.94123.75

[B30] Wira CR Fahey JV A new strategy to understand how HIV infects women: identification of a window of vulnerability during the menstrual cycle AIDS (London, England) 2008 22 15 1909 10.1097/QAD.0b013e3283060ea4 PMC2647143 18784454

[B31] Duffy L Culture and context of HIV prevention in rural Zimbabwe: the influence of gender inequality J Transcult Nurs 2005 16 1 23 31 10.1177/1043659604270962 15608096

[B32] Buvé A Bishikwabo-Nsarhaza K Mutangadura G The spread and effect of HIV-1 infection in sub-Saharan Africa Lancet 2002 359 9322 2011 2017 10.1016/S0140-6736(02)08823-2 12076570

[B33] UN Women out loud: How women living with HIV will help the world ends AIDS, in United Nations Joint Programme on HIV/AIDS 2012 Geneva, Switzerland: Joint United Nations Program on HIV/AIDS (UNAIDS)

[B34] Mfecane S Living with HIV as a man: implications for masculinity Psychol Soc 2008 36 45 59

[B35] Montgomery CM Men’s involvement in the South African family: engendering change in the AIDS era Soc Sci Med 2006 62 10 2411 2419 10.1016/j.socscimed.2005.10.026 16300871

[B36] Maleche A Day E Traditional cultural practices and HIV: reconciling culture and human rights Working Paper for the Third Meeting of the Technical Advisory Group of the Global Commission on HIV and the Law, 7–9 July 2011 2011 http://kelinkenya.org/wp-content/uploads/2010/10/TRADITIONAL-CULTURAL-PRACTICESHIV-RECONCILING-CULTURE-AND-HUMAN-RIGHTS.pdf

[B37] Hilber AM Intravaginal practices, vaginal infections and HIV acquisition: systematic review and meta-analysis PLoS One 2010 5 2 e9119 10.1371/journal.pone.0009119 20161749 PMC2817741

[B38] Samuels F Blake C Akinrimisi B HIV vulnerabilities and the potential for strengthening social protection responses in the context of HIV in Nigeria 2012 London, UK: The Overseas Development Institute

[B39] Clark S Bruce J Dude A Protecting young women from HIV/AIDS: the case against child and adolescent marriage International Family Planning Perspectives 2006 32 2 79 88 10.1363/3207906 16837388

[B40] Wand H Ramjee G The relationship between age of coital debut and HIV seroprevalence among women in Durban, South Africa: a cohort study BMJ Open 2012 2 e000285 22223838 10.1136/bmjopen-2011-000285 PMC3253418

[B41] Gillespie S Kadiyala S Greener R Is poverty or wealth driving HIV transmission? Aids 2007 21 S5 S16 18040165 10.1097/01.aids.0000300531.74730.72

[B42] Piot P Greener R Russell S Squaring the circle: AIDS, poverty, and human development PLoS Med 2007 4 10 e314 10.1371/journal.pmed.0040314 PMC2039763 17958469

[B43] Vandepitte J Estimates of the number of female sex workers in different regions of the world Sex Transm Infect 2006 82 Suppl 3 iii18 iii25 16735288 10.1136/sti.2006.020081 PMC2576726

[B44] WHO Preventing HIV among sex workers in sub-Saharan Africa: a literature review 2011 Geneva: WHO

[B45] Pruss-Ustun A HIV due to female sex work: regional and global estimates PLoS One 2013 8 5 e63476 10.1371/journal.pone.0063476 23717432 PMC3662690

[B46] Schwandt M Anal and dry sex in commercial sex work, and relation to risk for sexually transmitted infections and HIV in Meru, Kenya Sex Transm Infect 2006 82 5 392 396 10.1136/sti.2006.019794 16790563 PMC2563859

[B47] Luseno WK Substance use, sexual risk, and violence: HIV prevention intervention with sex workers in Pretoria AIDS Behav 2006 10 2 131 137 10.1007/s10461-005-9036-8 16482408

[B48] Coates TJ Richter L Caceres C Behavioural strategies to reduce HIV transmission: how to make them work better Lancet 2008 372 9639 669 684 10.1016/S0140-6736(08)60886-7 18687459 PMC2702246

[B49] Pettifor AE Sexual power and HIV risk, South Africa Emerg Inf Dis 2004 10 11 1996 10.3201/eid1011.040252 PMC3328992 15550214

[B50] Glynn JR Age at menarche, schooling, and sexual debut in northern Malawi PLoS One 2010 5 12 e15334 10.1371/journal.pone.0015334 21151570 PMC3000342

[B51] Greenberg J Magder L Aral S Age at first coitus a marker for risky sexual behavior in women Sex Transm Dis 1992 19 6 331 334 10.1097/00007435-199211000-00006 1492259

[B52] Manning WD Longmore MA Giordano PC The relationship context of contraceptive use at first intercourse Family Planning Perspectives 2000 32 3 104 110 10.2307/2648158 10894255

[B53] Boileau C Sexual and marital trajectories and HIV infection among ever-married women in rural Malawi Sex Transm Infect 2009 85 Suppl 1 i27 i33 10.1136/sti.2008.033969 19307337 PMC2654116

[B54] Pettifor AE Early age of first sex: a risk factor for HIV infection among women in Zimbabwe Aids 2004 18 10 1435 1442 10.1097/01.aids.0000131338.61042.b8 15199320

[B55] Laga M To stem HIV in Africa, prevent transmission to young women Aids 2001 15 7 931 934 10.1097/00002030-200105040-00014 11399966

[B56] Asiimwe-Okiror G Change in sexual behaviour and decline in HIV infection among young pregnant women in urban Uganda Aids 1997 11 14 1757 1763 10.1097/00002030-199714000-00013 9386811

[B57] Mah TL Halperin DT Concurrent sexual partnerships and the HIV epidemics in Africa: evidence to move forward AIDS Behav 2010 14 1 11 16 10.1007/s10461-008-9433-x 18648926

[B58] Potts M Reassessing HIV prevention Science 2008 320 5877 749 10.1126/science.1153843 18467575 PMC3501984

[B59] Karim SS Ramjee G Anal sex and HIV transmission in women Am J Public Health 1998 88 8 1265 1266 9702169 10.2105/ajph.88.8.1265-a PMC1508299

[B60] Lane T Heterosexual anal intercourse increases risk of HIV infection among young South African men Aids 2006 20 1 123 125 10.1097/01.aids.0000198083.55078.02 16327330

[B61] Simbayi LC Risk factors for HIV-AIDS among youth in Cape Town, South Africa AIDS Behav 2005 9 1 53 61 10.1007/s10461-005-1681-4 15812613

[B62] Scorgie F Socio-demographic characteristics and behavioral risk factors of female sex workers in sub-saharan Africa: a systematic review AIDS Behav 2012 16 4 920 933 10.1007/s10461-011-9985-z 21750918

[B63] Murphy EM Was the “ABC” approach (abstinence, being faithful, using condoms) responsible for Uganda’s decline in HIV? PLoS Med 2006 3 9 e379 10.1371/journal.pmed.0030379 17002505 PMC1564179

[B64] Gupta GR Structural approaches to HIV prevention Lancet 2008 372 9640 764 775 10.1016/S0140-6736(08)60887-9 18687460

[B65] Gupta GR How men’s power over women fuels the HIV epidemic: It limits women’s ability to control sexual interactions Br Med J 2002 324 7331 183 10.1136/bmj.324.7331.183 11809629 PMC1122116

[B66] Jewkes RK Levin JB Penn-Kekana LA Gender inequalities, intimate partner violence and HIV preventive practices: findings of a South African cross-sectional study Soc Sci Med 2003 56 1 125 134 10.1016/S0277-9536(02)00012-6 12435556

[B67] Leclerc-Madlala S Age-disparate and intergenerational sex in southern Africa: the dynamics of hypervulnerability Aids 2008 22 S17 S25 19033752 10.1097/01.aids.0000341774.86500.53

[B68] Serwadda D The social dynamics of HIV transmission as reflected through discordant couples in rural Uganda Aids 1995 9 7 745 750 10.1097/00002030-199507000-00012 7546420

[B69] Higgins JA Hoffman S Dworkin SL Rethinking gender, heterosexual men, and women’s vulnerability to HIV/AIDS Am J Public Health 2010 100 3 435 445 10.2105/AJPH.2009.159723 20075321 PMC2820057

[B70] Kim J Exploring the role of economic empowerment in HIV prevention Aids 2008 22 S57 S71 19033756 10.1097/01.aids.0000341777.78876.40

[B71] Jewkes RK Intimate partner violence, relationship power inequity, and incidence of HIV infection in young women in South Africa: a cohort study Lancet 2010 376 9734 41 48 10.1016/S0140-6736(10)60548-X 20557928

[B72] Campbell JC The intersection of intimate partner violence against women and HIV/AIDS: a review Int J Inj Contr Saf Promot 2008 15 4 221 231 10.1080/17457300802423224 19051085 PMC3274697

[B73] Vyas S Watts C How does economic empowerment affect women’s risk of intimate partner violence in low and middle income countries? A systematic review of published evidence J Int Dev 2009 21 5 577 602 10.1002/jid.1500

[B74] Poundstone K Strathdee S Celentano D The social epidemiology of human immunodeficiency virus/acquired immunodeficiency syndrome Epidemiol Rev 2004 26 1 22 35 10.1093/epirev/mxh005 15234945

[B75] Pulerwitz J Reducing HIV-related stigma: lessons learned from Horizons research and programs Public Health Rep 2010 125 2 272 281 20297756 10.1177/003335491012500218 PMC2821857

[B76] Lurie MN The impact of migration on HIV-1 transmission in South Africa: a study of migrant and nonmigrant men and their partners Sex Trans Dis 2003 30 2 149 156 10.1097/00007435-200302000-00011 12567174

[B77] McGowan I Rectal microbicides: a new focus for HIV prevention Sex Transm Infect 2008 84 6 413 417 10.1136/sti.2008.031328 19028937

[B78] Van Damme L Effectiveness of COL-1492, a nonoxynol-9 vaginal gel, on HIV-1 transmission in female sex workers: a randomised controlled trial Lancet 2002 360 9338 971 977 10.1016/S0140-6736(02)11079-8 12383665

[B79] Skoler-Karpoff S Efficacy of Carraguard for prevention of HIV infection in women in South Africa: a randomised, double-blind, placebo-controlled trial Lancet 2008 372 9654 1977 1987 10.1016/S0140-6736(08)61842-5 19059048

[B80] Van Damme L Lack of effectiveness of cellulose sulfate gel for the prevention of vaginal HIV transmission N Engl J Med 2008 359 5 463 472 10.1056/NEJMoa0707957 18669425

[B81] Abdool Karim Q Effectiveness and safety of tenofovir gel, an antiretroviral microbicide, for the prevention of HIV infection in women Science 2010 329 5996 1168 1174 10.1126/science.1193748 20643915 PMC3001187

[B82] Marrazzo J Pre-exposure prophylaxis for HIV in women: daily oral tenofovir, oral tenofovir/emtricitabine, or vaginal tenofovir gel in the VOICE Study (MTN 003) In 20th Conference on Retroviruses and Opportunistic infections 2013 Atlanta, USA: 2013 CROI conference

[B83] Network, M.T. MTN 020. 09 October 2013 Available from: http://www.mtnstopshiv.org/studies/3614

[B84] Baeten JM Antiretroviral prophylaxis for HIV prevention in heterosexual men and women N Engl J Med 2012 367 5 399 410 10.1056/NEJMoa1108524 22784037 PMC3770474

[B85] Van Damme L Preexposure prophylaxis for HIV infection among African women N Engl J Med 2012 367 5 411 422 10.1056/NEJMoa1202614 22784040 PMC3687217

[B86] Cohen MS Prevention of HIV-1 infection with early antiretroviral therapy N Engl J Med 2011 365 6 493 505 10.1056/NEJMoa1105243 21767103 PMC3200068

[B87] Grabbe KL Increasing access to HIV counseling and testing through mobile services in Kenya: Strategies, utilization and cost-effectiveness J Acquir Immune Defic Syndr 2010 54 3 317 10.1097/QAI.0b013e3181ced126 20453819 PMC3225204

[B88] Padian NS HIV prevention transformed: the new prevention research agenda Lancet 2011 378 9787 269 278 10.1016/S0140-6736(11)60877-5 21763938 PMC3606928

[B89] Medley A Effectiveness of peer education interventions for HIV prevention in developing countries: a systematic review and meta-analysis AIDS Educ Prev 2009 21 3 181 206 10.1521/aeap.2009.21.3.181 19519235 PMC3927325

[B90] Luchters S Impact of five years of peer-mediated interventions on sexual behavior and sexually transmitted infections among female sex workers in Mombasa, Kenya BMC public health 2008 8 1 143 10.1186/1471-2458-8-143 18445258 PMC2397398

[B91] Jewkes R Impact of stepping stones on incidence of HIV and HSV-2 and sexual behaviour in rural South Africa: cluster randomised controlled trial Br Med J 2008 337 a506 doi:10.1136/bmj.a506 10.1136/bmj.a506 18687720 PMC2505093

[B92] Bertrand JT Systematic review of the effectiveness of mass communication programs to change HIV/AIDS-related behaviors in developing countries Health Educ Res 2006 21 4 567 597 16847044 10.1093/her/cyl036

[B93] Van Rossem R Meekers D The reach and impact of social marketing and reproductive health communication campaigns in Zambia BMC Public health 2007 7 1 352 10.1186/1471-2458-7-352 18088437 PMC2245935

[B94] Keating J Meekers D Adewuyi A Assessing effects of a media campaign on HIV/AIDS awareness and prevention in Nigeria: results from the VISION Project BMC Public health 2006 6 1 123 10.1186/1471-2458-6-123 16672067 PMC1508144

[B95] Shelton JD Partner reduction is crucial for balanced “ABC” approach to HIV prevention Br Med J 2004 328 7444 891 10.1136/bmj.328.7444.891 15073076 PMC387490

[B96] Dworkin SL Blankenship K Microfinance and HIV/AIDS prevention: assessing its promise and limitations AIDS Behav 2009 13 3 462 469 10.1007/s10461-009-9532-3 19294500 PMC3770268

[B97] Parker RG Easton D Klein CH Structural barriers and facilitators in HIV prevention: a review of international research Aids 2000 14 S22 S32 10981471 10.1097/00002030-200006001-00004

[B98] Gregson S Community group participation: Can it help young women to avoid HIV? An exploratory study of social capital and school education in rural Zimbabwe Soc Sci Med 2004 58 11 2119 2132 10.1016/j.socscimed.2003.09.001 15047071

[B99] Jukes M Simmons S Bundy D Education and vulnerability: the role of schools in protecting young women and girls from HIV in southern Africa Aids 2008 22 S41 S56 19033754 10.1097/01.aids.0000341776.71253.04

[B100] Slutkin G How Uganda reversed its HIV epidemic AIDS Behav 2006 10 4 351 360 10.1007/s10461-006-9118-2 16858635 PMC1544374

